# Temporal turnover and the maintenance of diversity in ecological assemblages

**DOI:** 10.1098/rstb.2010.0285

**Published:** 2010-11-27

**Authors:** Anne E. Magurran, Peter A. Henderson

**Affiliations:** 1 School of Biology, University of St Andrews, St Andrews, Fife KY16 8LB, UK; 2 Pisces Conservation Ltd, IRC House, The Square, Pennington, Lymington SO41 8GN, UK

**Keywords:** biodiversity, turnover, estuarine, fish, abundance, rank

## Abstract

Temporal variation in species abundances occurs in all ecological communities. Here, we explore the role that this temporal turnover plays in maintaining assemblage diversity. We investigate a three-decade time series of estuarine fishes and show that the abundances of the individual species fluctuate asynchronously around their mean levels. We then use a time-series modelling approach to examine the consequences of different patterns of turnover, by asking how the correlation between the abundance of a species in a given year and its abundance in the previous year influences the structure of the overall assemblage. Classical diversity measures that ignore species identities reveal that the observed assemblage structure will persist under all but the most extreme conditions. However, metrics that track species identities indicate a narrower set of turnover scenarios under which the predicted assemblage resembles the natural one. Our study suggests that species diversity metrics are insensitive to change and that measures that track species ranks may provide better early warning that an assemblage is being perturbed. It also highlights the need to incorporate temporal turnover in investigations of assemblage structure and function.

## 1. Introduction

Growing concern about the state of the planet and the impacts that we humans are having on it (Butchart *et al*. 2010) has led to renewed interest in methods used to track changes in biodiversity, which is generally done in one of two ways (Magurran *et al*. in press). The first approach is to combine information on the status of individual species to provide an overview of change, as, for example, the Living Planet Index (Loh *et al*. 2005) does. These types of indicators are popular with politicians and policy-makers and are increasingly widely used, but can have the disadvantage that conclusions are made on the basis of a non-random selection of species. Alternatively, one can look at the assemblage as a whole and monitor changes in biodiversity in relation to baseline changes—that is in comparison with the extent of temporal shifts in species identities and species abundances that would occur in an unperturbed assemblage.

Assemblage-based assessments track these shifts in an ecologically coherent group of organisms, and as such provide a link between biodiversity and ecosystem function (Cardinale *et al*. 2007, 2009; Loreau 2010). Temporal variation—turnover—in species abundances (Srivastava & Vellend 2005; Loreau 2010) can help maintain function if there is asynchrony in species abundances such that species that decline are replaced with those that have similar functional roles. Two closely related hypotheses—the ‘portfolio effect’ (Doak *et al*. 1998; Tilman *et al*. 1998) and the ‘insurance hypothesis’ (Yachi & Loreau 1999)—argue that ecosystem properties are more stable in diverse assemblages because there will be more species to compensate for any changes that do occur.

Although an assemblage-wide assessment of biodiversity is appealing, there are a number of difficulties. First, apart from a few well-known examples, such as the Park Grass experiment (Lawes & Gilbert 1880; Lawes *et al*. 1882) and the Continuous Plankton Recorder (Richardson *et al*. 2006), there are relatively few long-term datasets in which biodiversity data have been collected using consistent methods over a number of decades (Wolfe *et al*. 1987; Magurran *et al*. in press)—but this is now changing (e.g. Ernest *et al*. 2008). Second, although it is well known that all ecological communities experience temporal turnover (Darwin 1859; MacArthur & Wilson 1967), we still have limited information on how assemblage biodiversity changes through time (but see Grossman *et al*. 1982; Sale & Douglas 1984; Ross *et al*. 1985; Warwick *et al*. 2002; Thibault *et al*. 2004; Pavoine *et al*. 2009 for some exceptions). This arises not just as a consequence of data shortage but also because models of species abundance have focused on spatial rather than temporal patterns (Magurran 2007). As a result, it can be difficult to know whether reported changes in biodiversity are greater than those that might have happened by chance.

In general, it seems that mature assemblages—that is, those not undergoing any directional change such as succession—tend to show high persistence in the sense that species richness is maintained through time (Ross *et al*. 1985). This constancy can be underlain by considerable temporal variation in species rank and species abundance (Grossman *et al*. 1982, 1990; Ross *et al*. 1985), and thus is consistent with the idea that temporal asynchrony in species abundances can help stabilize community properties (Loreau 2010). However, Cottingham *et al*. (2001) found that the relationship between species richness and the regulation of aggregate assemblage variables (such as biomass and productivity) was more complex than anticipated. Moreover, it appears that species abundances do not always vary asynchronously. Valone & Barber (2008), for instance, discovered that the abundances of most pairs of species in an analysis of a range of terrestrial assemblages tended to covary positively—and attributed this to correlated responses to climatic fluctuations. Houlahan *et al*. (2007) found that the abundances of species in natural communities tended to covary positively and argued that the absence of compensatory dynamics is because abiotic factors, including climate, are likely to be more important than competition. On the other hand, Leary & Petchey (2009) showed that experimental communities of protists that contained species with different responses to environmental changes exhibited lower temporal variation in biomass. It is clear that our knowledge of the natural pattern of change in ecological assemblages is incomplete, and to address this, we need to examine data that have been collected using consistent methods, at regular intervals and ideally over decades so that we can be sure that natural variation is accounted for. Two factors warrant investigation: (i) do species abundances in natural assemblages vary asynchronously and (ii) which patterns of asynchrony (i.e. turnover in species abundances) produce assemblages where the structure is consistent with that found in nature and where assemblage properties are maintained through time.

In this paper, we explore temporal changes in an exceptionally well-documented assemblage of estuarine fishes and compare these patterns with those generated by a simulated fish assemblage with the same set of species, in which we manipulate the degree of within-species autocorrelation in abundance across years. We initially focus on the empirical dataset and begin by asking whether the abundances of the individual species change asynchronously, as predicted by the insurance and portfolio hypotheses discussed above. Next, we relate this pattern to assemblage structure with the expectation that this structure is preserved through time (i.e. that there will be no trend in a range of diversity metrics). Then, using the simulated data, we examine the conditions under which the aggregated abundances of the individual species produce an assemblage that resembles the natural one. We do this because while asynchrony in species abundances may be necessary to assure continuity of assemblage properties, year-to-year changes in the abundance of species will be constrained by their ecology, with the result that only a limited range of turnover scenarios are likely to be present in natural systems. For this analysis, we assume that the abundances of species in the assemblage vary independently, as each oscillates around its historical mean. Thus, we are asking how the correlation between the abundance of a species in a given year and its abundance in the previous year influences the structure of the overall assemblage. We assess assemblage responses to these varying levels of autocorrelation by using diversity measures that evaluate assemblage structure in terms of the relative abundances of species, but ignore species identities, and with metrics that track individual species identities. As Whittaker (1960) recognized, measures that take account of species composition can uncover differences between assemblages that indices that examine species richness or species relative abundance might not pick up (see also Grossman *et al*. 1982). We, therefore, expect the metrics that track species identities to reveal a smaller set of turnover scenarios in which the predicted assemblage resembles the real one.

## 2. Methods

### (a) Data collection

The Bristol Channel estuarine fish assemblage has been sampled monthly for 30 years (Henderson 2007; Henderson & Bird 2010). To date, greater than 80 species and greater than 100 000 individuals have been recorded.

Fish samples were collected from the cooling water filter screens at Hinkley Point B power station, situated on the southern bank of the Bristol Channel in Somerset, England. The water intakes are in front of a rocky promontory within Bridgwater Bay, and to the east are the 40 km^2^ Stert mudflats. Depending upon the tide, the fish were sampled from water varying in depth from about 8 to 18 m. For a full description of the intake configuration and sampling methodology, see Henderson & Holmes (1991) and Henderson & Seaby (1994). The filter screens have a solid square mesh of 10 mm. Methodology has not changed over the three decades of the study.

Quantitative sampling commenced in 1980 when 24 h surveys of the diurnal pattern of capture were undertaken in October and November. From these surveys, it was concluded that samples collected during daylight were representative of the 24 h catch (Henderson & Holmes 1990) and monthly quantitative sampling commenced in January 1981. The total volume of water sampled per month, which has not varied over the entire period, is 4.27 × 10^5^ m^3^. To standardize for tidal influence, all sampling dates were chosen for tides halfway between springs and neaps, with sampling commencing at high water (normally about 12.00 h). Fish were collected hourly from two filter screens for a 6 h period, identified to species and the number of individuals recorded.

The power station intakes at Hinkley Point are an effective sampler because of their position at the edge of a large inter-tidal mudflat in an estuary with extremely powerful tides resulting in suspended solid levels of up to 3 g l^−1^ and little light below 50 cm depth. The fishes, pelagic or benthic, are moved towards the intake in the tidal stream, often as they retreat from the inter-tidal zone where they feed, and it is likely that they are unable to see or otherwise detect the intake until they are too close to make an escape. Light is clearly important for avoidance because at power station intakes situated in clear water captures are higher at night. The efficiency of the sampling method is discussed in Henderson & Holmes (1991).

### (b) Data analysis

We ask whether species abundances vary asynchronously by examining pairwise correlations, through time, for both the core assemblage (i.e. species that are present in majority of years; Magurran & Henderson 2003) and the entire assemblage (all species recorded). In both cases, we compare the empirical median correlation coefficient with the values generated by a randomization test (repeated 1000 times) in which the abundances of each species are randomly shuffled among years.

To examine the link between the patterns of autocorrelation and assemblage structure, we employ the following time-series model (Crawley 2007):
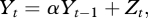

where *Y*_*t*_ is the abundance of each species at time *t*, *Y*_*t*__−1_ the abundance of species in the previous year, *α* the correlation structure (within species, between years) and *Z*_*t*_ the noise in year *t* (set by a random number generator with mean = 0 and s.d. = empirical value).

Starting values are set at 0 and the output rescaled by adding the empirical mean (for each species) to obtain the predicted values. The model is run for values of *α* between −1 (strong anti-correlations) and +1 (random walk) in steps of 0.05. In each case, the autocorrelation value is equal for all species, while noise is independent for each species. Moreover, there is no among-species correlation included in the model, and species dynamics are completely independent. This analysis is repeated 1000 times and for both the core and the entire assemblage. We run the model for 50 years and use the last 30 years in our analyses. While we appreciate that strong negative correlations in the abundance of these estuarine fishes—many of which are relatively long lived—are biologically unlikely, we include these in our analysis to provide an insight into the consequences of these extreme values for temporal trends in assemblage diversity.

We assess the structure of the resulting assemblages by using three measures that place different weights on the relative abundance of species. These measures, which are well-known diversity statistics (Magurran 2004) and numerically related to one another (Hill 1973), are the exponential form of the Shannon index (*N*_1_ in Hill's terminology), the reciprocal form of the Simpson index (*N*_2_) and the Berger–Parker index (*N*_∞_). The exponential form of the Shannon index emphasizes the species richness component of diversity, the reciprocal form of the Simpson index emphasizes dominance, whereas the Berger–Parker index is a pure measure of dominance (specifically the relative abundance of the most abundant species). Together they provide an informative overview of the structure of the assemblage.

These diversity measures, however, ignore species identities. To track change in composition, we use two metrics, mean rank shift (MRS) and Bray–Curtis dissimilarity. MRS (Collins *et al*. 2008) is a measure of relative change in species rank abundance. The Bray–Curtis dissimilarity is a widely used measure of ecological distance (Southwood & Henderson 2000; Magurran 2004; Jost *et al*. in press) that assesses compositional similarity taking account of species abundances. Whereas the Hill numbers provide a value of diversity for each year of the series, MRS and Bray–Curtis dissimilarity make comparisons between consecutive pairs of years in the time series.

To compare the natural and simulated patterns, we first calculate the 95 per cent confidence limits for a given metric using the annual values in the empirical time series. We then take the mean predicted value for each level of autocorrelation and average these across the last 30 years of the simulated series (as above). These predicted values are then plotted in relation to the 95 per cent confidence limits of the empirical values (figure 4) as a simple method of examining the agreement between the observed and expected diversity metrics.

All analyses use the statistical programming language R (R Development Core Team 2008). Hill's diversity numbers and Bray–Curtis dissimilarity were calculated using the vegan (Oksanen 2010) package.

## 3. Results

**Figure 1.**
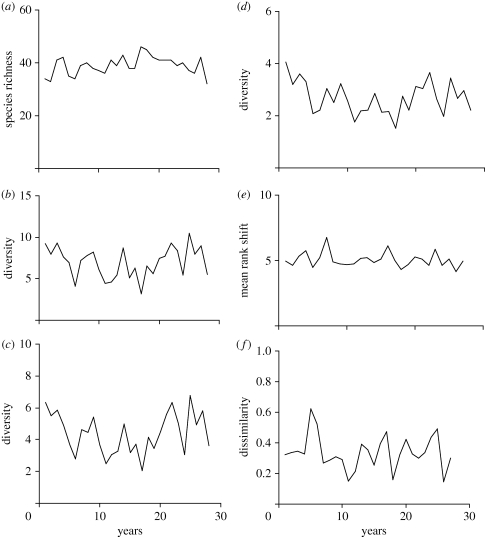
Change in the Hinkley fish community through time: overall community view. (*a*) Species richness per year of time series (Hill's *N*_0_ index); (*b*) exponential form of the Shannon index (Hill's *N*_1_ index), annual values; (*c*) reciprocal of Simpson diversity index, annual values; (*d*) Berger–Parker index (Hill's *N*_∞_ index), annual values; (*e*) MRS index, sequential pairs of years; (*f*) Bray–Cutis dissimilarity values between sequential pairs of years. The different metrics exhibit year-to-year variation but in no case is there a significant linear trend: (*a*) *y* = 0.090*x* + 37.61, *R*^2^ = 0.045; (*b*) *y* = 0.006*x* + 6.90, *R*^2^ = 0.0007; (*c*) *y* = 0.0031*x* + 4.35, *R*^2^ = 0.004; (*d*) *y* = 0.015*x* + 2.91, *R*^2^ = 0.039; (*e*) *y* = −0.012*x* + 5.22, *R*^2^ = 0.03; (*f*) *y* = −0.0014*x* + 0.356, *R*^2^ = 0.009.

**Figure 2.**
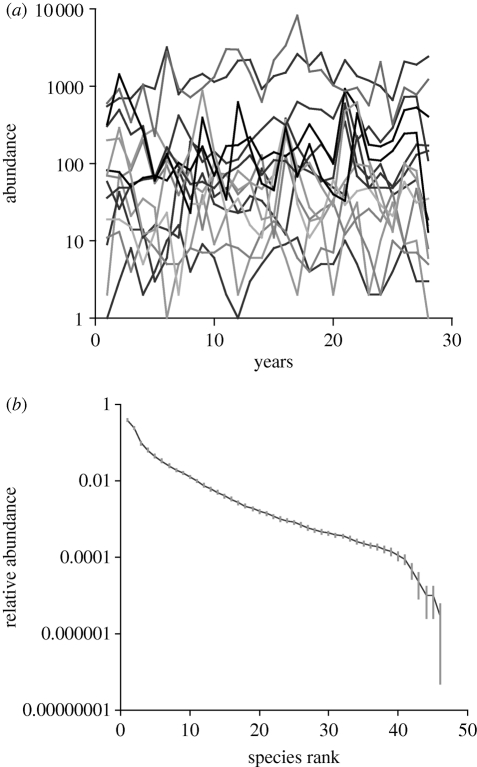
(*a*) Temporal variation in the abundance of the 15 species that are present in every year of the time series; (*b*) rank abundance plot where values for first-ranked species disregarding species identity, second-ranked species, third-ranked species, etc. have been averaged across the time series. Grey bars show the 95% confidence limits around these mean values.

The structure of this estuarine assemblage has remained essentially unchanged through the study period (figure 1). This is true for the core species, as well as for the assemblage as a whole. Although some of our metrics show year-to-year variation, in no case is there a significant linear trend in the time series. This constancy at the assemblage level is maintained despite substantial changes in abundance of individual species, as figure 2*a* clearly reveals. The conservation of the assemblage's structure (which is also apparent in figure 2*b*) is not a product of temporal cross-correlations (either positive or negative) in species abundances. Pairwise correlations computed among species (through years) for the core assemblage (Magurran & Henderson 2003), and the entire assemblage, do not appear to differ from chance in a biologically meaningful way. The median correlation coefficient (Pearson's *r*) for the empirical dataset (core species) is −0.019. The 2.5 and 97.5 per cent quantiles (based on the randomization test) are −0.048 and −0.018, respectively. Equivalent values for the entire assemblage are −0.057 (quantiles −0.060 and −0.053).

There is thus no strong signal of cross-correlation (either positive or negative) in species abundances through time. This outcome, which confirms that changes in the abundances of the species in the assemblage are effectively asynchronous, is consistent with a recent investigation of the dynamics of the dominant species in a range of vertebrate and invertebrate communities (Mutshinda *et al*. 2009). (Mutshinda *et al*. included a sub-set of our dataset in their analysis and reached a very similar conclusion.) This is not to say that no species pairs are correlated through time. For example, the abundances of two co-generic taxa, the pout, *Trisopterous luscus*, and poor cod, *T. minutus*, both rise and fall in concert, but show only modest temporal correlation (*r* = 0.31, *n* = 28) because they periodically switch their relative abundances. There are also instances of correlations in the abundances of closely related species in other communities (Sugihara *et al*. 2003).

**Figure 3.**
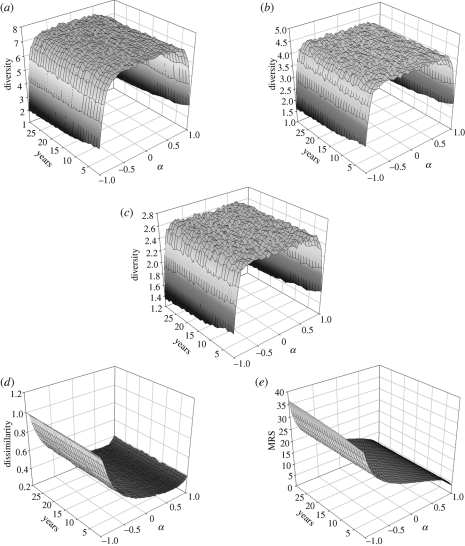
Predicted values of (*a*) *N*_1_, exp Shannon index, (*b*) *N*_2_, 1/Simpson index, (*c*) *N*_∞_, Berger–Parker index, (*d*) Bray–Curtis dissimilarity and (*e*) MRS over 30 years in relation to varying values of *α*.

**Figure 4.**
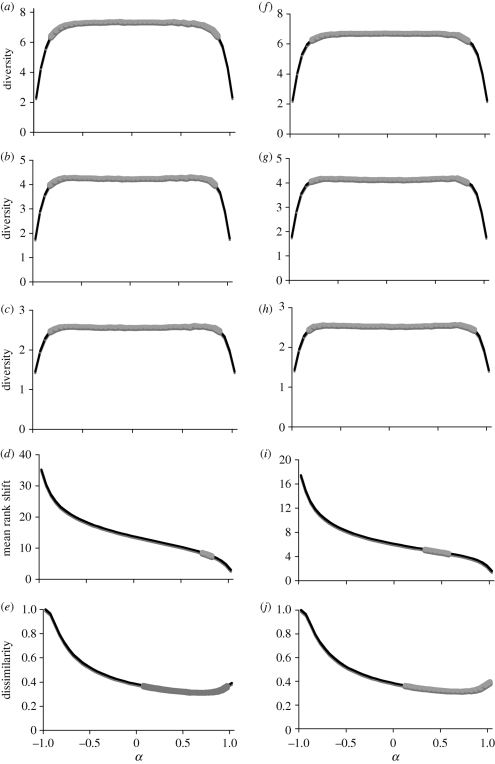
Relationship between community characteristics (diversity (*a*–*c*, *f*–*h*) and community composition (*d*,*e*,*i*,*j*)) and correlation structure (*α*) for the entire community (*a*–*e*) and the core community (*f*,*j*). Graphs plot predicted values (mean of 30*y*). Points that lie within the 95% confidence limit of the empirical value are indicated by the thicker grey line. (*a*,*f*) *N*_1_, exp Shannon index, (*b*,*g*) *N*_2_, 1/Simpson index, (*c*,*h*) *N*_∞_, Berger–Parker index, (*d*,*i*) MRS and (*e*,*j*) Bray–Curtis index.

Our model predicts temporal patterns of species abundances that are consistent with the observed values. When *α* is around 0 (i.e. where the abundance of a species in one year is uncorrelated with its abundance in the previous year, resembling white noise) the results are similar to the empirical pattern. When *α* approaches −1 (strong anti-correlations) or *α* approaches 1 (a classic random walk) predicted abundances begin to diverge from the observed values. This result is particularly striking when we examine the 30 year trends of the Hill diversity numbers. In each case, and across a broad range of autocorrelation values, the same level of diversity is maintained through time (figure 3*a*–*c*). Moreover, when −0.8 < *α* < 0.8, predicted mean diversity falls within the confidence limits of the empirical values (figure 4*a*–*c*, *f*–*h*). This is true for both the core assemblage and the entire assemblage. In other words, the structure of the assemblage is retained as long as the autocorrelation in abundances between successive years is not too strong.

In contrast, metrics that take species identities into account reveal that the empirical assemblage is replicated over a narrower range of conditions. While both Bray–Curtis dissimilarity and MRS remain constant through time for a given value of *α* (figure 3*d*,*e*), the relationship between the extent of autocorrelation and the predicted value of the metric is an asymmetric one. As figure 4*d*,*e* and *i*,*j* indicates, only positive values of *α* lead to predictions that lie within the empirical confidence limits. This is particularly evident for MRS. Here, the predicted core assemblage resembles the empirical one when *α* ≈ 0.5 (figure 4*i*). The still narrower band of matching MRS values for the entire assemblage (figure 4*d*) is due to the occasional species which shift between being present at a low rank and being absent (Magurran & Henderson 2003).

## 4. Discussion

Our results show that abundances of species in an estuarine assemblage of fishes change asynchronously through time. We compared this pattern with a model that varied the level of autocorrelation in species abundances and used three informative diversity measures to describe different aspects of the species abundance distribution. Our model was able to replicate empirical patterns of diversity, not just in terms of the predicted level of diversity but also its constancy through time, for each of these three measures. Furthermore, this result was repeated for a wide range of autocorrelation values. In other words, we found that there are many different ways in which species abundances can change asynchronously and still produce an assemblage that is indistinguishable—based on diversity indices—from the natural one.

Our model incorporates MacArthur's (1960) prediction that species typically return to their equilibrium level following a rise or decline in abundance. We are not making any assumption about whether the assemblage as a whole is in equilibrium here, but simply reflecting the fact that in the natural assemblage species tend to vary around their mean abundances. Because these changes in species abundances occur asynchronously, they are also consistent with the portfolio effect, as well as with the insurance hypothesis and its expectation that niche differences will cause species to respond to environmental variations in different ways and with different lags (Yachi & Loreau 1999; Loreau 2010). However, we have not formally explored the compensatory dynamics hypothesis (Tilman *et al*. 1998) which argues that ecosystem function is preserved as a result of negative covariance in species abundances and is in any case not well supported by empirical data (Houlahan *et al*. 2007). Although we have not shown that niche differences in this estuarine assemblage cause this asynchronicity, we have evidence that niche differences exist and underpin the relative abundances of species (Magurran & Henderson 2003; Henderson & Magurran 2010). Interestingly, the maintenance of structure through time is to a large extent due to the presence of the core species (figure 2*b*)—which are also the most common ones and contribute in excess of 98 per cent of the overall abundance in any given year (Magurran & Henderson 2003). It is increasingly recognized that common species make a significant contribution to assemblage structure and function (Gaston & Fuller 2008) and this is borne out by our work. On the other hand, the richness of the assemblage is maintained by the presence of the infrequent species; these predominantly rare species contribute a constant fraction of the overall richness of the assemblage through time (Magurran & Henderson 2003).

The conclusions we reach about the maintenance of assemblage structure using diversity measures contrast with those obtained with metrics that take account of species identities. In these latter cases, there is a more limited set of circumstances in which temporal turnover produces patterns that replicate those seen in nature. Thus, while the structure of the assemblage, in terms of the relative abundances of species, appears insensitive to variation in the rate of temporal turnover, there is a much smaller set of conditions that generate realistic year-by-year changes in species rankings and species composition.

An intriguing finding is that the metrics that track species composition suggest that the natural assemblage structure is generated by a positive autocorrelation of around 0.5. This value implies that the community metric has some predictability and the time series is dominated by longer wavelengths and has some memory or inertia to change. In fact, the reddening of population time series is a commonly observed feature. Kaitala *et al*. (2001) argue that reddened or pink population time series are a product of spatial structure and migration between populations. This must certainly be the case for the Hinkley data where there is a continual influx of migrants (Magurran & Henderson 2003). In addition to the role of metapopulation dynamics in reddening time series, we need also to consider the role of reddened environmental variables. Almost all physical time series influencing fishes at Hinkley, such as sea water temperature, solar radiation and North Atlantic Oscillation, have pink spectra. Finally, it is important to note that all longer-lived species have populations with memory. The number of offspring produced in any year is not just a product of events at reproduction but events over a number of previous years that have influenced the quality and abundance of the adults. This is the inertia or memory that can produce pink noise. It is therefore possible that an autocorrelation of around 0.5 is an emergent property of a community which has (i) some memory (the number of a species next year is linked to past reproductive success), (ii) metapopulation dynamics that tend to produce slower, longer-term changes, and (iii) a response to environmental variables with pink spectra.

A large number of metrics have been developed to assess the changes that occur in ecological communities as a result of human disturbance. A significant fraction of these have focused on changes in the species abundance distribution. For example, it has been suggested that an intact assemblage will have a lognormal pattern of species abundances, whereas an impacted one will be closer to a log-series distribution (May 1975). Unfortunately empirical data reveal a wide range of assemblage responses (Gray 1987; Tokeshi 1993; Dornelas *et al*. 2010). We suggest that because species abundance distributions are unlikely to change as long as the species richness of the assemblage is preserved, classical diversity measures may not be sensitive measures of disturbance. Like Collins *et al*. (2008) and MacNally (2007), we conclude that other indicators, such as those that track species ranks or changes in species composition, may provide better early warning that the assemblage is entering a transitional state, or has already been impacted.

Our results remind us that comparisons between the observed and expected distributions of relative abundances of species in an ecological assemblage will not necessarily be the best way of distinguishing between competing mechanistic models (McGill *et al*. 2007). We also note that we still have a very incomplete understanding of how ecological communities change through time. There have been numerous attempts (e.g. Motomura 1932; May 1975; Tokeshi 1996; Harte *et al*. 1999; Hubbell 2001; McGill 2003) to account for the characteristic shape of species abundance distributions. However, although some of the pioneers in the field (notably Fisher *et al*. 1943; Preston 1960) included temporal dynamics in their models, most attention has been directed to spatial patterns (but see Rosenzweig 1995; Lande *et al*. 2003; Magurran & Henderson 2003; Thibault *et al*. 2004 for some exceptions). These findings suggest that models that track temporal changes need to account for shifts in species composition as well as changes in species abundance. We anticipate that this new generation of models will become increasingly important in monitoring and managing changes in biodiversity.

Finally, we note that species complementarity—one of the explanations for increased productivity in diverse communities (Tilman 1997; Tilman *et al*. 1997, 2001; Hector *et al*. 1999)—has been shown to become increasingly important with time (Loreau *et al*. 2003; Cardinale *et al*. 2007; Fargione *et al*. 2007). The complementarity hypothesis argues that there are niche differences which mean that species exploit the available resources in different, and complementary, ways (Fargione *et al*. 2007). However, rapid changes in abundance could reduce complementarity and impair function. We agree with Schwartz *et al*. (2000) that the rate of temporal turnover in species abundances may play a critical role in ecosystem functioning and deserves more study. Temporal turnover may also be a variable that needs to be taken into account when assessing the capacity of natural communities to exhibit resilience in the face of environmental change (Thrush *et al*. 2009).
